# Green Labelled Rice Shows a Higher Nutritional and Physiochemical Quality Than Conventional Rice in China

**DOI:** 10.3390/foods10050915

**Published:** 2021-04-21

**Authors:** Jiuliang Xu, Jiahui Zhong, Baozhen Zhang, Xuexian Li

**Affiliations:** 1 The Key Plant-Soil Interaction Laboratory, Department of Plant Nutrition, Ministry of Education, China Agricultural University, Beijing 100193, China; jlxu9@cau.edu.cn (J.X.); zhongjiahuicau@126.com (J.Z.); zhangBzzzZ@163.com (B.Z.); 2 National Academy of Agriculture Green Development, China Agricultural University, Beijing 100193, China; 3 Chinese Academy of Green Food Development, Beijing 100193, China

**Keywords:** chinese green food, rice, physicochemical properties, elements, metabolomics

## Abstract

In China, green food refers to a wide array of certified agricultural and processed edible commodities that are produced strictly following defined standard protocols and labelled with a specified “Green Food” logo. The demand for green labelled rice is rapidly growing due to its higher quality and adherence to safety standards compared to conventional rice. Therefore, the physicochemical and nutritional quality of green rice needs to be further investigated for consumers’ benefits. Using Daohuaxiang 2, one of the most famous types of green rice, we found that green rice was significantly superior to conventional rice in terms of thousand kernel weight, chalkiness, amylose content, and rheological properties. Green rice contained lower levels of heavy metals than conventional rice due to a dramatic reduction in chemical inputs during its cultivation. The concentrations of Cr, As, Cd, Pb in green rice decreased, respectively, from 98.7 to 180.1 μg/kg, 49.8 to 62.3 μg/kg, 7.8 to 9.1 μg/kg, and 29.0 to 42.8 μg/kg on average. Gas chromatography coupled with mass spectrometry (GC–MS)-based metabolomics, in combination with multivariate analysis, revealed that 15 metabolites differentially accumulated when comparing green and conventional rice. Among these, 12 metabolites showed a high accumulation in green rice, including seven amino acids, two sugars, and three fatty acids. Overall, our results suggest the superior quality of a type of green rice that is popular in China, which may boost green rice consumption and facilitate the further expansion of green rice production in China.

## 1. Introduction

Rapid economic growth in China has been accompanied by the increasing concern of consumers with respect to food quality and safety. In China, there are three levels of certification systems for food, which is indicative of the stringency of the associated standards, namely safe food, green food, and organic food [1]. Green food was first introduced by the Ministry of Agriculture of China in 1990, and it refers to a unique Chinese certification scheme for food, which indicates that the food was produced in accordance with the principle of sustainable development and that standard operational protocols were applied throughout the whole industry chain, as designated by the China Green Food Development Center (CGFDC) [2]. Green food, which is of a relatively lower standard compared with organic food, provides a “middle way” between safe and organic food and is widely accepted in China [3]. At the end of 2019, China already had 13,202 green-certified enterprises, representing 36,345 products and 21.7 million farmers, with 11.1 million hectares being used for the production of 157 million tons of green products. The annual sales and export values were Chinese Yuan (RMB) 455.7 billion yuan and USD 3.21, accounting for 8.20% of the total farmland area in China, and 9.7% of the GDP was from agriculture [4].

Rice is one of the most important staple cereal foods for half of the world’s population. In order to ensure food security, many countries frequently apply greater amounts of chemical fertilizers than required. The average rate of applied nitrogen (N) fertilizer was 180 kg N/ha for rice in China, which is 75% higher than the world average [5]. The application of excessive nitrogen fertilizer is a serious problem in China, which can not only decrease the grain yield and quality, but has also caused serious environmental problems [6,7,8]. Nitrogen fertilization is the most important factors for effectively improving rice yield and quality [9,10,11]. Recently, many optimized nutrient management strategies have been applied in China to improve rice yield and quality [12,13,14]. The combined application of an organic fertilizer with a chemical fertilizer is an important approach for increasing fertilizer use efficiency and soil microbial activity, which can improve the yield and quality of rice [15,16]. In the specific case of green-certified production, the application of chemical nitrogen fertilizer has been reduced by 50% compared with local farmers, and its use in combination with organic fertilizers is encouraged, since they are better able to meet the quality and safety demands of customers [4].

Rice comprises the largest proportion of green food products and accounts for 14.9%. According to statistics, the yearly output yield of green-certified rice is 15.6 million tons, which was 7.4% of the total output of the nation in 2019 (CGFDC). The most important at-attitudinal factors relating to consumers’ choice of green food include health concerns, environmental concerns, and taste preferences [17]. To date, studies on green food have mainly focused on the environment, economic yields, and consumers’ willingness to pay [1,2,18,19,20]. Rice quality largely determines competitiveness in China. However, consumers are poorly informed about the quality of green food, though the difference in quality between green and conventional rice remains largely unclear. Thus, scientific evidence in support of the benefits of green foods is greatly needed to promote consumer confidence in such foods.

Wuchang is a city located in the northeast of China, and it is an important rice growing area with natural conditions that are excellent for this purpose. Wuchang rice has be-come extremely popular and well-known and is associated with high quality rice in China [21]. The brand Wuchang rice was valued at more than 69.8 billion yuan in 2020, ranking at number one among rice brands in China (National Bureau of Statistics). Daohuaxiang 2 (*Oryza sativa* ssp. japonica cv. Daohuaxiang 2) is predominantly grown in Wuchang city, Heilongjiang Province. Thus, Daohuaxiang 2 was selected for this study. The aim of the present study was to investigate and compare the nutritional quality of green labelled (hereafter referred to as “green rice”) and conventional rice. To the best of our knowledge, no papers comparing the quality of green and conventional rice have been published. This paper reports the first study investigating the effects of green versus conventional agricultural practices on rice quality. It is expected that the information obtained in the present study could provide insights into green food in China and help consumers be better informed about green food, thus, promoting consumer confidence.

## 2. Materials and Methods

### 2.1. Samples

Twenty commercially available Daohuaxiang 2 rice samples, including ten green-certified and ten conventional rice samples, were purchased from a local Beijing supermarket. According to the selection criteria, the same variety (Daohuaxiang 2) and representative brands available at the time of sampling were selected as the experimental materials (Supplementary Materials Table S1). All of the green rice samples were certified by the China Green Food Development Center. The rice samples were ground into fine powder and sieved through a 55-mesh count screen, and the powder was stored at −80 °C until use.

### 2.2. Chemicals and Reagents

High-performance liquid chromatography (HPLC)-grade methanol, acetonitrile, dichloromethane, and water were obtained from Fisher Scientific (Pittsburgh, PA, USA). Formic acid of HPLC grade was purchased from TIC (Tokyo, Japan). Bis (trimethylsilyl) trifluoroacetamide (BSTFA), trimethylchlorosilane (TMCS), adonitol, nitric acid, acetic acid, and perchloric acid were obtained from Sigma Aldrich (St. Louis, MO, USA).

### 2.3. Physicochemical and Rheological Properties

The thousand kernel weight (TKW) of green and conventional rice was determined in triplicate based on randomly selected kernels that were weighed using an electrical balance. The bulk density was calculated as the ratio of milled rice grains to their volume and is reported as g/mL. The chalkiness degree was assessed according to the method of the China National Standard NY/T 593–2013. The moisture, protein, and fat content were analyzed by near-infrared (NIR) spectroscopy (FOSS-NIRSDS 2500, FOSS Analytical, Hoganas, Sweden). The determination of amylose was carried out calorimetrically using the amylose–iodine reaction. The amylose content was calculated using mixed standards of amylose and amylopectin [22]. The peak viscosity, final viscosity, breakdown viscosity, setback viscosity, peak time, and pasting temperature were measured using a Rapid Visco Analyzer (Perten RVA 4500, Segeltorp, Sweden) to evaluate the rheological properties of the starch structure, according to the manufacturer’s instructions.

### 2.4. Elemental Analysis

The elements in rice were determined using the method described in a previous report [23]. In brief, 500 mg rice samples were placed in a polytetrafluoroethylene (PTFE) digestion container. Nitric acid (6 mL) was added to each container and predigested overnight at 60 °C. Hydrogen peroxide (2 mL) was added after cooling. The heating program was performed in three steps. In the first step, the temperature was increased linearly to 120 °C over a period of 6 min and maintained for 2 min. In the second step, the temperature was increased linearly to 160 °C (1500 W) over a period of 4 min and maintained for 8 min. For the third step, the temperature was increased linearly to 180 °C (1500 W) over a period of 3 min and maintained for 20 min, followed by 30 min of cooling. The essential elements in the rice were determined by inductively coupled plasma-atomic emission spectrometry (ICP–AES) using the Optima 7300DV (Perkin Elmer, Waltham, CO, USA), and the toxic elements were determined using a 7700 Series × ICP–MS (Agilent Technologies, Courtaboeuf, France).

### 2.5. Non-Targeted Metabolomics Analysis

Metabolomic analysis using gas chromatography coupled with mass spectrometry (GC-MS) was performed according to a previous report, with slight modifications [24]. First, 100 mg rice powder was added into 1.0 mL water–methanol–dichloromethane solution (1:3:1) and 40 μL internal standard solution (200 μg/mL, adonitol). The extraction was carried out by sonication for 30 min, and the mixture was then centrifuged for 10 min at 16,000× *g*. Next, 0.7 mL of the upper layer solution was transferred to a 1.5 mL Eppendorf tube and vacuum-dried in a CentriVap centrifugal vacuum concentrator (Labconco, Kansas City, MO, USA). Sixty microliters of methoxyamine hydrochloride (20 mg/mL in pyridine) was added to each vial and incubated for 30 min at 80 °C. Subsequently, 80 μL BSTFA reagent (1% TMCS, *v*/*v*) was added, followed by a reaction time of 1.5 h at 70 °C. GC-MS analysis was conducted using an Agilent 7890 gas chromatograph (Agilent Technologies, Palo Alto, CA, USA) system equipped with a Shimadzu GC–MS QP2010 Plus (Shimadzu, Kyoto, Japan). The instrumental settings followed previous protocols [24].

### 2.6. Data Processing and Multivariate Data Analysis

Tentative identification of metabolites was achieved using National Institute of Standards and Technology (NIST) 15 standard mass spectral databases (NIST, Gaithersburg, MD, USA) with a similarity of more than 80%, verified by available reference compounds. Peak areas were normalized to the internal standard and exported to MS Office Excel (version 2019, Microsoft Corporation., Redmond, WA, USA). Principal component analysis (PCA) and partial least squares-discriminant analysis (PLS-DA) was conducted using SIMCA-P + software (version 14.0, Umertrics, Umea, Sweden). Statistical analysis was conducted using the SPSS statistical software (version 21; SPSS Corp., Chicago, IL, USA). The Student’s *t*-test was performed to analyze significance of cross-group difference, and *p* values lower than 0.05 indicated statistical significance.

## 3. Results and Discussion

### 3.1. Physicochemical and Rheological Properties

#### 3.1.1. Physical Properties

The physical properties, including the thousand kernel weight, bulk density, chalkiness, and percentage of chalky kernels, of the green and conventional rice are reported in Table 1. The thousand kernel weight of rice is an important parameter for the evaluation of rice yield, and green rice showed a significantly higher thousand kernel weight (21.08 g) compared to that of conventional rice (20.21 g). No significant difference in bulk was observed between the green and conventional rice groups. Chalky rice kernels usually cause low eating quality and strongly influence consumer acceptance of products [25]. Green rice had a relatively lower chalkiness (8.31%) and percentage of chalky kernels (22.46%) than conventional rice (9.74% and 26.15%, respectively).

Most inspection guides for rice use chalkiness and the percentage of chalky kernels for grading. The chalky grain rate of first-grade high-quality rice is lower than 10%, while that of inferior rice is higher than 30% in China [26]. Chalkiness is a complex polygenic trait, and nitrogen fertilizers had significant effects on the chalky trait of rice grain. Many studies have reported that nitrogen fertilization significantly increased the percentage of chalky kernels and chalkiness [9,27,28]. Thus, the chalky trait of rice from different production systems may be due to the management of different fertilizers. The adoption of green food production systems, with combined application of organic and inorganic fertilizers, is advisable as a good compromise for chalky rice [6].

#### 3.1.2. Main Chemical Components

The main chemical components of green and conventional rice are shown in Table 1. Protein content is a crucial factor in rice quality. In the present study, green rice exhibited a lower protein content (7.11 g per 100 g) in comparison to conventional rice (7.42 g per 100 g). Amylose content is also an important factor for estimating the cooking or eating quality of rice. Table 1 shows that the amylose content in green rice was higher than in conventional rice. Moisture and fat contents greatly affect the quality of rice. In this study, the results showed no significant difference in moisture and fat contents between the green and conventional rice.

Previous studies have reported that the protein content is negatively correlated to the palatability of rice [29,30,31]. Many studies have reported that a high protein content is associated with a harder cooked rice and may influence its eating quality [32,33]. Similar findings have been reported in an organic rice cropping system, with organic rice showing a lower accumulation of protein than conventional rice [34,35]. It has been observed that the application of a nitrogen fertilizer significantly increases the protein content of rice [27,36]. Previously, a study reported that the combined application of 40% organic fertilizer and 60% chemical fertilizer was the optimal ratio for obtaining the best quality of rice [37].

Low-amylose rice is generally soft and sticky after cooking, whereas high-amylose rice is harder and less sticky. This result, in accordance with previous results, indicate that organic rice shows a higher amylose content than conventional rice [34,38]. A higher amylose content may be associated with fertilizer application. Iqbal et al. reported that the use of an organic fertilizer combined with a chemical fertilizer considerably improved the rice amylase content [39].

#### 3.1.3. Rheological Properties

The pasting properties, as determined by RVA, are widely accepted indicators for the critical and rapid assessment of the eating and cooking quality of rice [40]. The peak viscosity, final viscosity, breakdown viscosity, and setback viscosity of the green rice and conventional rice are shown in Table 2. The present study shows that the pasting properties of the green rice and conventional rice were significantly different (*p* < 0.05). Peak viscosity is an indicator of water-binding capacity. The peak viscosity of green rice ranged from 3625 to 4463 centipoise (cP), with a mean value of 4250 cP, while for conventional rice, this ranged from 3417 to 4325 cP, with a mean value of 4055 cP. The final viscosity indicates the ability of the starch to obtain a gel structure after cooking and cooling. The final mean viscosity value of 4353 cP for green rice was high compared to that of conventional rice. Breakdown viscosity is associated with the ease of cooking rice starch, and this value for green rice (1526 cP) was higher than that of conventional rice (1478 cP), but this difference was statistically insignificant (*p* = 0.5231). Setback viscosity indicates the rate of starch retrogradation, and green rice was found to have lower rates of starch retrogradation compared with conventional rice.

Cooking quality is an important attribute with respect to consumers’ acceptance, and has been found to vary significantly for rice depending on the cultivation system. Overuse of N fertilizers leads to poor eating and cooking quality of the grain [12]. The pasting properties were negatively correlated with the protein content [41]. The green rice with good taste values showed a higher peak viscosity and final viscosity, by contrast, with a lower setback viscosity. A higher peak, final, and setback viscosity was reported in organic rice compared to the conventional product [35,41]. Nonetheless, our study suggested that the cooking and eating quality of green rice was significantly improved due to the adoption of green rice management practice.

### 3.2. Elemental Signatures of Green and Conventional Rice

#### 3.2.1. Toxic Elements

**Figure 1:**
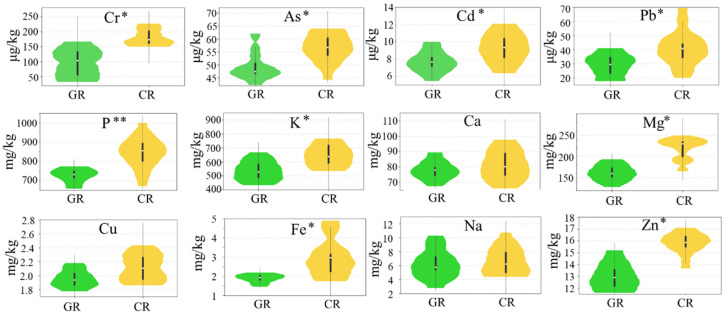
Violin plot of toxic elements and essential elements in the green rice (GR) and conventional rice (CR) systems (* *p* < 0.05, ** *p* < 0.005).

Four toxic elements of the rice samples were analyzed (Figure 1). Chromium (Cr) is considered a serious environmental pollutant due to its wide industrial applications. Evidence has demonstrated that Cr can pose serious health risks to humans, causing diarrhea, asthma, and cancer [42]. The Cr concentration in the green rice (98.7 μg/kg) was lower than that in the conventional rice (180.1 μg/kg), and the green rice ranged from 18.1–170.7 μg/kg, while the range in the conventional rice was 150.4–226.3 μg/kg.

Arsenic (As) is a ubiquitous metalloid that can cause cancer in humans and enter the food chain mainly from contaminated drinking water [43]. Rice has been reported to contain a more toxic form of arsenic in comparison to other plants [44]. The As content of green rice (42.2–60.7 μg/kg) was lower than that of the conventional rice (44.4–88.9 μg/kg). The As content in this study can be considered low in comparison with that reported in other studies [45,46].

Rice grains around the world are frequently contaminated with cadmium (Cd), which poses a serious threat to human health and has attracted widespread concern [47]. The concentration of Cd in green rice is lower than that in conventional rice. Soil conditions and management practices can significantly influence the Cd content in rice. Previous studies have indicated that the application of organic fertilizer significantly reduces the Cd content in rice plants [48]. Green food reduces chemical nitrogen fertilizer use by 50% compared with the fertilization levels local farmers through supplementation with organic fertilizers [4]. The limit for Cr in rice established by the Ministry of Agriculture is 200 μg/kg. Both types of rice showed very low levels of Cd content, which were well below the prescribed limit.

Lead (Pb) is one of the more frequently studied elements in rice [44,49]. It has been reported that the mean concentration of Pb in rice collected in China is 100 ± 140 μg/kg [50]. In this study, Pb was quantified at concentrations ranging from 17.6 to 40.9 μg/kg for green rice and from 20.1 to 80.6 μg/kg for conventional rice, values which are within the Ministry of Agriculture’s maximum permissible limit for this metal (200 μg/kg).

#### 3.2.2. Essential Elements

The levels of eight essential elements (P, K, Ca, Mg, Cu, Fe, Na, and Zn) were determined in the rice samples. These micronutrients are important for human health, and a deficiency of these microelements can result in serious diseases. The ANOVA results of each element for the green and conventional rice showed that the P, K, Mg, Fe, and Zn levels were significantly different (*p* values < 0.05). P and K were the most abundant essential elements in both types of rice, and the P contents of the green rice and conventional rice ranged from 657.3 to 772.0 mg/kg and 671.3 to 995.2 mg/kg, respectively. For K, the content found in green rice ranged from 453.8 to 667.4 mg/kg, which is lower than that in the conventional rice, which ranged from 535.1 to 739.5 mg/kg. Both elements were significantly higher in conventional rice, which may be due to the fact that P and K are contained in synthetic fertilizer and are largely used in conventional rice farming.

Mg is an essential element for the human body. Many studies have reported that an Mg deficiency is associated with a wide range of diseases, including cardiovascular diseases, arrhythmia, osteoporosis, and fibromyalgia [51,52]. The Mg content of green rice (128.1–192.7 mg/kg) was lower that of conventional rice (165.7–248.4 mg/kg). A previous study reported synergistic behavior between the Mg and K concentrations [53].

The iron content of green rice ranged from 1.5 to 2.2 mg/kg, while the range for conventional rice was between 1.7 to 4.9 mg/kg. Previous studies have reported that the application of a nitrogen fertilizer promoted Fe accumulation in rice grain [54]. Iron deficiency was recently listed as a public health problem and the 6th leading cause of disease in developing countries by the World Health Organization (WHO) [55]. The amount of bioavailable iron in rice is low, and the phytic acid present in rice may inhibit the bioavailability of iron [56].

Likewise, the zinc (Zn) concentrations in green rice ranged from 11.6 to 15.2 mg/kg, while the concentration in conventional rice ranged from 13.7 to 17.1 mg/kg. The mean zinc content in green rice was 13.3 mg/kg, and the mean value in conventional rice was 15.3 mg/kg. Zn is one of the most essential elements required for the growth of human beings. The relatively low Zn concentrations found in both types of rice cannot meet the daily dietary requirement [57].

The results of the present study indicate the importance of management practice in contributing to rice element accumulation. Concentrations of all examined toxic (Cr, As, Cd, and Pb) and essential elements (P, K, Mg, Fe, and Zn) in green rice were lower compared to those of conventional rice. Chemical fertilizers promote the absorption of more elements from the soil by the rice. There were no significant differences found in Ca, Cu, and Na between the green and conventional rice. The synthetic N, P, and K compound fertilizers are applied at high concentrations in conventional rice, so the associated elements are accumulated at higher levels. With strict regulations and regular inspection, green rice contained significantly lower levels of toxic elements than conventional rice. These results are useful as scientific evidence of the greater safety of green rice in comparison with conventional rice in terms of toxic metal levels. In a study by Xiao et al. in China, the range of Cr, As, Cd, and Pb contents in rice increased from 0 to 200 μg/kg, 100 to 200 μg/kg, 0 to 150 μg/kg, and 0 to 100 μg/kg, respectively [58]. When comparing these results with ours (Figure 1), all of the toxic elements are well within the acceptable range. Indeed, Cd was much lower than the average in China, which may be due to the low Cd accumulation in the soil in northern China compared to the other areas [59]. Additionally, the content of toxic elements in both types of rice was well below the prescribed limit, indicating that the twenty brands of rice sold in Beijing markets can be considered as very safe. Consumer demand for healthier and safer rice that contains a lower content of toxic elements is growing. In our study, green rice was demonstrated to be safer than conventional rice. However, it is also worth noting that the levels of essential elements were observed to be lower in green rice than in conventional rice, suggesting that greater efforts should be made toward improving the content of essential elements in green rice by biofortification in the future.

### 3.3. Untargeted Metabolomics

**Figure 2:**
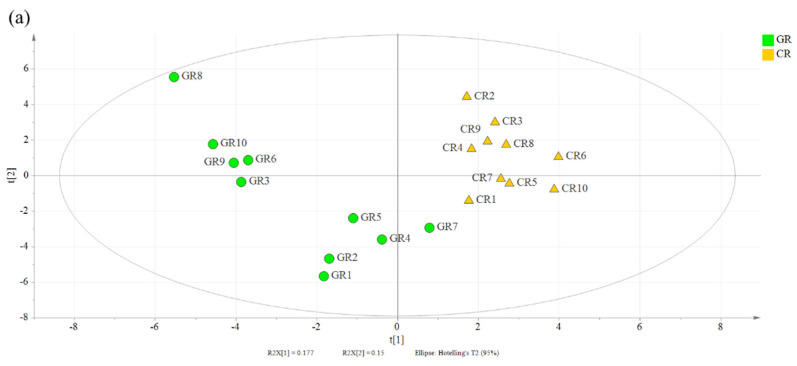

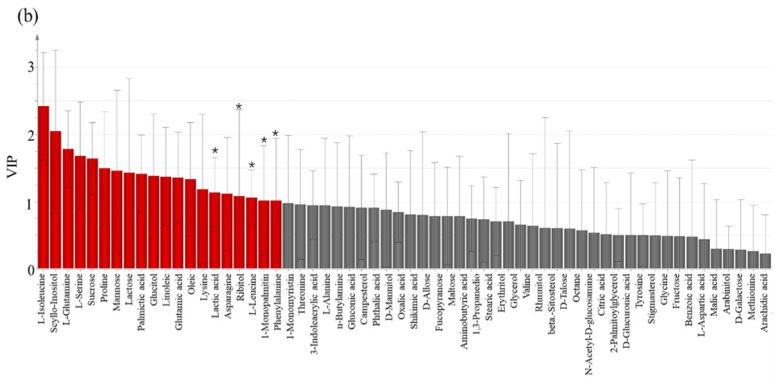
Partial least squares-discriminant analysis (PLS-DA) score plot (**a**) of primary metabolites identified by GC–MS. (**b**) Metabolites (**b**) that were differentially expressed between GR (green rice) and CR (conventional rice) with a VIP > 1.0 were considered significant. * denotes *p* > 0.05 (insignificant).

Untargeted metabolomics was used to provide comprehensive information for comparing green rice and conventional rice. In this study, the metabolites from rice grains were identified by GC–MS. Metabolite identification was performed through a comparison of the results with authentic standards, fragmentation patterns, and database references. In total, 64 metabolites, including amino acids, organic acids, sugars, sugar alcohols, and amines, were identified in the rice samples. The relative intensities were normalized based on the intensities of the internal standards and were used to perform multivariate statistical analyses. PCA and PLS-DA were used to analyze the metabolomics datasets for green and conventional rice. The PCA score plots exhibited clear differentiations between the green and conventional rice for PC1 (28.1%) and PC2 (12.2%) (Supplementary Materials Figure S1). A similar separation pattern was observed using PLS-DA, including 23.8% of the first and 15.3% of the second principal components (Figure 1), and 15 significantly different metabolites were selected, with a variable importance for projection (VIP) > 1.0 and *p* < 0.05 (Figure 2). These significant differing metabolites served as characteristic compounds that could be used for differentiating green rice and conventional rice.

**Figure 3:**
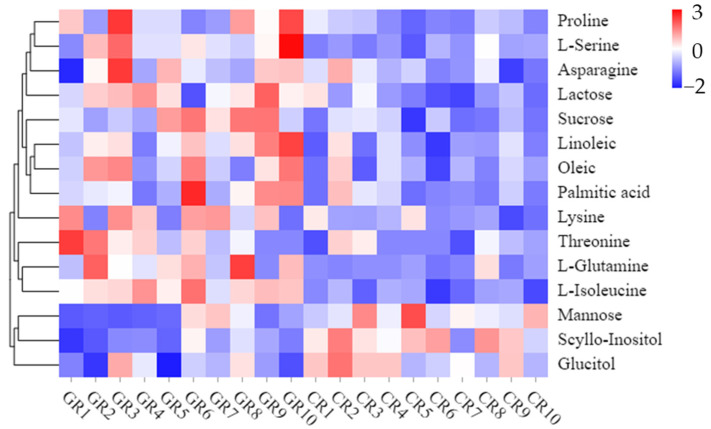
Heatmap of fifteen key metabolites differentially expressed between green and conventional rice. Note: GR = green rice, CR = conventional rice. The more intense red color indicates a higher level of metabolite expression; the more intense blue color indicates a lower level of metabolite expression.

The 15 differential metabolites included seven amino acids, five sugars, and three fatty acids. The heatmap shows the abundance of metabolites in different clusters from each sample (Figure 3). In green rice, seven (proline, serine, asparagine, lysine, threonine, L-glutamine, and L-isoleucine) out of 18 amino acids accumulated to significantly higher levels than in conventional rice. Sugars are important for the taste and quality of the grain. Sucrose and lactose were found to be highly expressed in green rice. In contrast, higher levels of myo-inositol, mannose, and glucitol were found in green rice compared to the conventional rice. Three fatty acids, including oleic, linoleic, and palmitic acid, were significantly differentially expressed between the green and conventional rice, with expression higher in green rice.

The results of untargeted metabolomics in this study revealed that the metabolic variations between green and conventional rice were significantly different. Amino acids are essential nutritional components in rice. The difference in amino acid contents between green and conventional rice may be due to their different production systems. The green rice was produced with a limited amount of chemical fertilizers in combination with organic fertilizers. In a previous study, the combined application of organic and chemical fertilizers significantly increased the nitrogen use efficiency, soil matter, microbial activity in the soil, and rice yield [15,16,60]. Significantly increased total essential amino acid contents have also been found when using combined chemical and organic fertilizers [61,62]. However, organic rice has shown a lower or similar level of amino acids than conventional rice [63,64]. 15N-labeled tracer techniques showed that combined application of chemical and organic fertilizers significantly increased the percentages of organic N and amino acid N [65]. The release of N from organic fertilizers partially contributed to increased amino acid accumulation.

We noted that the contents of sucrose and lactose were significantly higher in green rice than in conventional rice, with *p*-values of less than 0.001. It was reported that nitrogen overfertilization significantly inhibited sugar biosynthesis and lower its accumulation with the application of K [66]. However, three other sugars, including myo-inositol, mannose, and glucitol, were present in higher amounts in conventional rice compared to green rice. The reason for this phenomenon remains unanswered at present. Oleic, linoleic, and palmitic acid are the major fatty acids, accounting for more than 90% of the total fatty acids in rice [67]. All of the three fatty acids were significantly higher in the green rice than in the conventional rice, and this is an important consideration for future research. Fatty acids are highly associated with fungi, and the addition of organic fertilizers significantly increased the fungal population [68]. It has been reported that these fatty acids contribute to the prevention of several diseases, such as cardiovascular disease, inflammatory disease, and cancer [69].

In our study, most metabolites were highly accumulated in green rice. Most probably, the production systems of green rice involved chemical fertilizers combined with organic fertilizers, which can improve the utilization rate of fertilizers and the microbial activities in soil and alter metabolic pathways to certain extent within the plant, thus, enhancing the accumulation of beneficial metabolites in rice.

## 4. Conclusions

Currently, the growing concern of consumers as regards food safety and the environment is gaining strength. The current agricultural system in China heavily depends on the use of chemical N, which negatively affects N use efficiency and soil health, the environment, and food safety. This paper investigated and compared the quality and metabolomics of rice grown in green-certified versus conventional production systems. The physicochemical and rheological properties were higher for rice grown under the green-certified system than under the conventional farming system. The toxic elements in both types of rice were very low, indicating that the investigated rice sold in Beijing markets can be regarded as very safe, although the green rice was found to be safer than the conventional rice, as it had a lower toxicity. However, the essential elements (P, K, Mg, Fe, and Zn) were present at lower levels in green rice compared with conventional rice. Through untargeted metabolomics analysis using GC–MS, we found that 15 of the 64 detected metabolites were more highly expressed in green rice, and three metabolites were more highly expressed in the conventional rice. The differences between the two types of rice in terms of metabolites may be mainly due to the reductions in the use of synthetic fertilizers and the combined use of organic and chemical fertilizers. Thus, the green rice production system is a potential alternative to the conventional system, which allows for a reduction in chemical fertilizer use, producing safer rice of a higher quality and, thus, is financially attractive to farmers. The results of this study could be useful in the further development of green rice production in China.

## Figures and Tables

**Table 1:** Physicochemical properties of rice grains produced by the green food and conventional systems.

Parameters	Green Rice	Conventional Rice
Thousand kernel weight (g)	21.08 ± 3.23 a	20.21 ± 3.11 b
Bulk density (g/mL)	0.98 ± 0.12 a	0.96 ± 0.09 a
Chalkiness (%)	8.31 ± 1.47 b	9.94 ± 2.26 a
Percentage of chalky kernels	22.46 ± 5.06 b	26.15 ± 3.43 a
Moisture content (g/100 g)	11.65 ± 0.23 a	11.53 ± 0.31 a
Protein (g/100 g)	7.11 ± 0.56 b	7.42 ± 0.71 a
Fat content (g/100 g)	0.80 ± 0.12 a	0.77 ± 0.13 a
Amylose content (g/100 g)	17.2 ± 0.82 a	16.3 ± 0.91 b

Values (± standard deviation) within the same row followed by “a” are significantly higher than those indicated by “b” (*p* < 0.05) (*n* = 3).

**Table 2:** Pasting properties of rice starch obtained from green rice (GR) and conventional rice (CR) systems.

Parameters	GR	CR
Peak viscosity (cP)	4250 ± 371 a	4055 ± 411 b
Final viscosity (cP)	4353 ± 324 a	4132 ± 321 b
Breakdown viscosity (cP)	1526 ± 142 a	1478 ± 156 a
Setback viscosity (cP)	1011 ± 179 b	1231 ± 165 a

Values (± standard deviation) within the same row followed by “a” are significantly higher than those indicated by ”b” (*p* < 0.05) (*n* = 3).

## Data Availability

Data is contained within the article or Supplementary Materials.

## Supplementary Materials

The following are available online at https://www.mdpi.com/article/10.3390/foods10050915/s1, Table S1: The information of rice samples from green rice and conventional rice. Figure S1: PCA score plots derived from non-targeted metabolite profiling of three different treatments analyzed by GC-MS.
