# Supplementary material for: Green Labelled Rice Shows a Higher Nutritional and Physiochemical Quality Than Conventional Rice in China
Source: Foods. 2021 Apr 21;10(5):915. doi: 10.3390/foods10050915 (PMC8143485; doi:10.3390/foods10050915)
Supplement: Supplementary file 1 [file foods-10-00915-s001.zip › foods-1187902-supplementary/Supplemental data.pdf]

Table S1. The information of rice samples from green rice and conventional rice.

Green rice				
	Brand	Price (Yuan/kg)	Longitude and latitude	Picture
1	Wu mi chang xiang	16.0	127.3004,44.9262	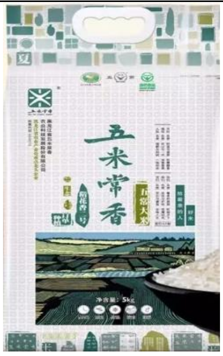
2	San liang lv zhu	27.8	126.9376, 45.1525	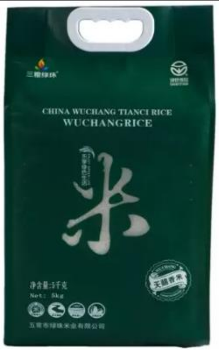
3	Dao ke dao	16.0	127.1125, 44.8952	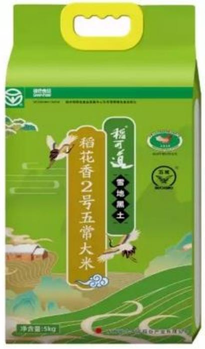
4	Tian fu	23.6	127.7456, 44.6632	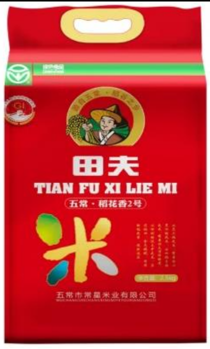

5	Chang wang	18.4	127.2089, 44.7058	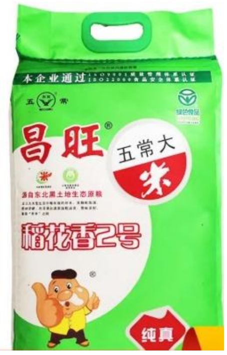
6	Longfengshan	17.6	127.5865, 44.7770	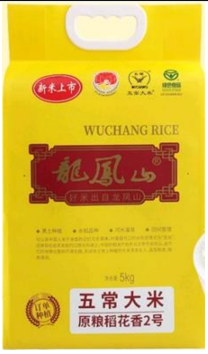
7	Liangshi	15.0	127.3869, 44.9111	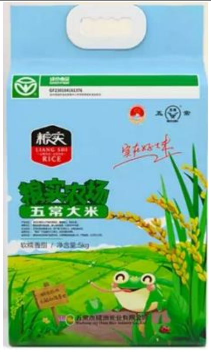
8	Qiaofudayuan	64.5	127.1748, 44.8721	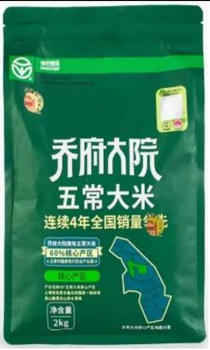
9	Heitubiji	31.6	127.1674, 44.9319	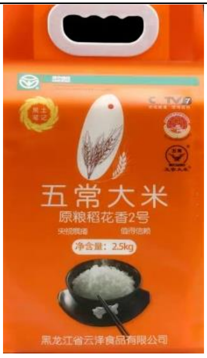

10	Tianciliangyuan	17.6	127.0853, 45.0548	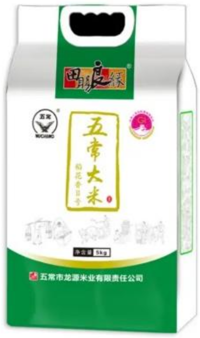

Conventional rice				
	Brand	Price (Yuan/kg)	Longitude and latitude	Picture
1	Xin you gu	8.6	127.5781, 44.7694	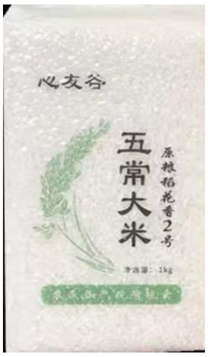
2	Wan xi feng shou ji	8.4	127.1525, 44.9152	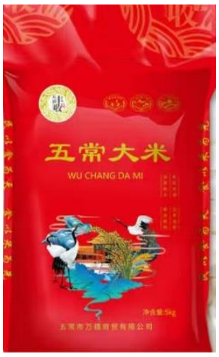
3	Ting xiang	6.4	127.1675, 44.9319	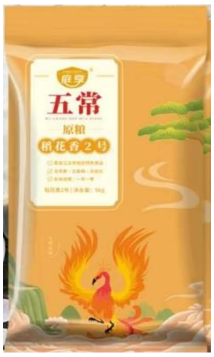

4	Jia he xiang	10.0	127.1675, 44.9319	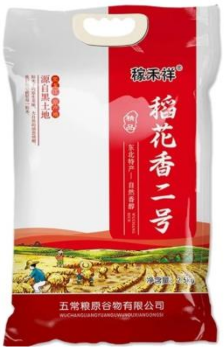
5	Shen xian dao	11.2	127.0958, 44.9144	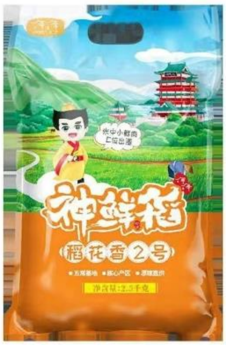
6	Shi zai ren	9.0	127.0958, 44.9144	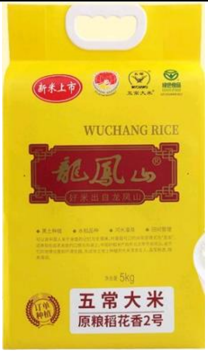
7	Zhong mi bei fang	11.6	126.8648, 45.2368	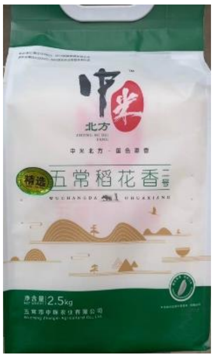

8	Sheng feng	6.8	127.1399, 44.9362	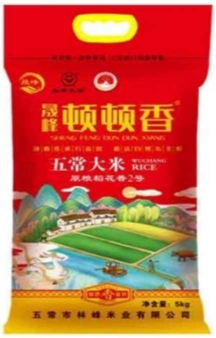
9	Xiangyan	7.0	127.2093, 44.7013	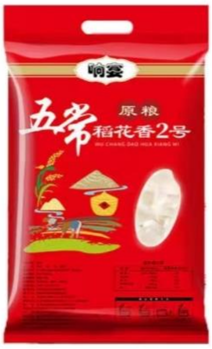
10	Nan ji ren	14.0	127.4846, 44.9098	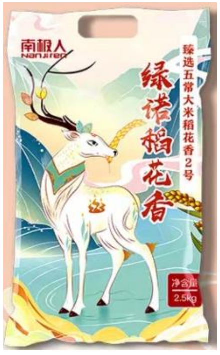

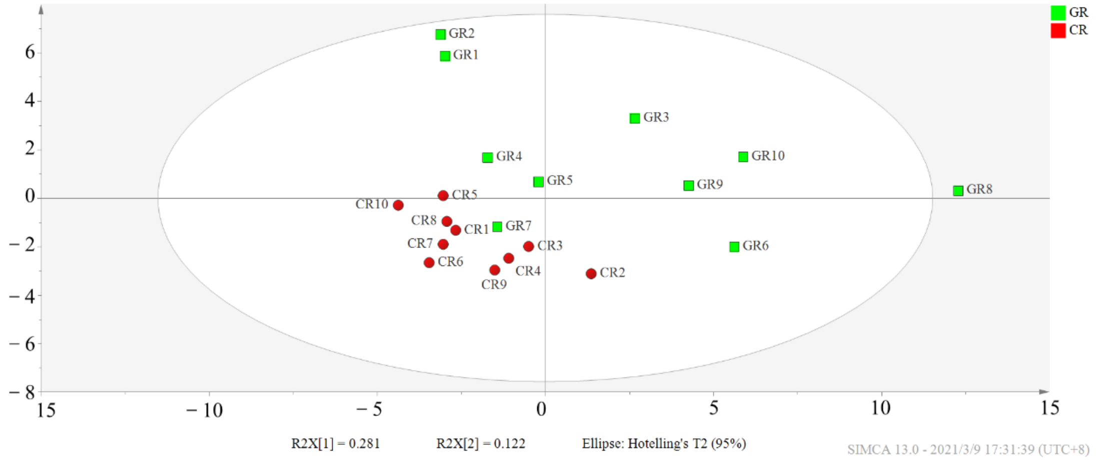

Figure S1. PCA score plots derived from non-targeted metabolite profiling of three different treatments analyzed by GC-MS.
